# Supplementary material for: Attendance, engagement and performance in a medical school curriculum: early findings from competency-based progress testing in a new medical school curriculum
Source: PeerJ. 2018 Jul 30;6:e5283. doi: 10.7717/peerj.5283 (PMC6071618; doi:10.7717/peerj.5283)
Supplement: Data S2 [file peerj-06-5283-s002.docx]

Description of Raw Data File in Excel format.

Categorized number of IRAT sessions missed

ES-F16-1-Total Percent Locally Developed Exam

ES-F16-2-Percent Score Locally Developed Exam

ES-S17-1- Total Percent Locally Developed Exam

ES-S17-2 Total Percent Locally Developed Exam

Total Score F16-1 NBME Customized Exam

Total Score 16-2 NBME Customized Exam

Total score 17-1 NBME Customized Exam

Total Score S17-2 NBME Customized Exam

PCSE FS16-1 Post Encounter

PCSE FS16-2 Post Encounter

PCSE SS17-1 Post Encounter

PCSE SS17-2 Post Encounter

Number of IRAT zeros (missed sessions)

Number of NBME scores 1 SD or more below the mean

Number of ExamSoft scores 1 SD or more below the mean

Combined numbe of ExamSoft and NBME scores 1 SD or more below the mean

Number of PCSE scores 1 SD below the mean

Categorized number of exams 1 SD below mean
